# Galectin-3 predicts response and outcomes after cardiac resynchronization therapy

**DOI:** 10.1186/s12967-018-1675-4

**Published:** 2018-11-03

**Authors:** Clémentine Andre, Eric Piver, Romain Perault, Arnaud Bisson, Julien Pucheux, Emmanuelle Vermes, Bertrand Pierre, Laurent Fauchier, Dominique Babuty, Nicolas Clementy

**Affiliations:** 1 Cardiology Department, Trousseau Hospital, University of Tours, 37044 Tours, France; 2 Biochemistry Department, Trousseau Hospital, University of Tours, Tours, France; 3 Imaging Department, Trousseau Hospital, University of Tours, Tours, France

**Keywords:** Galectin-3, Heart failure, Cardiac resynchronization therapy

## Abstract

**Background:**

Cardiac resynchronization therapy (CRT) reduces symptoms, morbidity and mortality in chronic heart failure patients with wide QRS complexes. However, approximately one third of CRT patients are non-responders. Myocardial fibrosis is known to be associated with absence of response. We sought to see whether galectin-3, a promising biomarker involved in fibrosis processes, could predict response and outcomes after CRT.

**Methods:**

Consecutive patients eligible for implantation of a CRT device with a typical left bundle branch block ≥ 120 ms were prospectively included. Serum Gal-3 level, Selvester ECG scoring, and cardiac magnetic resonance with analysis of late gadolinium enhancement (LGE) were ascertained. Response to CRT was defined by a composite endpoint at 6 months: no death, nor hospitalization for major cardiovascular event, and a significant decrease in left ventricular end-systolic volume of 15% or more.

**Results:**

Sixty-one patients were included (age 61 ± 5 years, ejection fraction 27 ± 5%), 59% with non-ischemic cardiomyopathy. At 6 months, 49 patients (80%) were considered responders. Responders had a lower percentage of LGE (8 ± 13% vs 22 ± 16%, p = 0.006), and a trend towards lower rates of galectin-3 (16 ± 6 ng/mL vs 19 ± 8 ng/mL, p = 0.13). LGE ≥ 14% and Gal-3 ≥ 22 ng/mL independently predicted response to CRT (OR = 0.17 [0.03–0.62], p = 0.007, and OR = 0.11 [0.02–0.04], p < 0.001, respectively). At 48 months of follow-up, 12 patients had been hospitalized for a major cardiovascular event or had died. Galectin-3 level predicted long-term outcomes (HR = 3.31 [1.00–11.34], p = 0.05).

**Conclusions:**

Gal-3 serum level predicts the response to CRT at 6 months and long-term outcomes in chronic heart failure patients.

## Background

Heart failure (HF) is a progressive disease still characterized by high morbidity and mortality. Cardiac resynchronization therapy (CRT) has a rightful place in the management of HF and is recommended by the European Society of Cardiology (ESC) [1, 2], as it dramatically reduces hospitalizations for HF and overall mortality [3, 4]. However, many factors influence the efficacy of CRT, leading to a 20–30% rate of so-called “non-responders” to this therapy [5]. One of the main predictors of non-response is the presence of intra-myocardial scar, especially when localized at the left ventricular (LV) pacing site [6, 7].

Cardiac magnetic resonance imaging (CMR) remains the gold standard to evaluate the extent of LV myocardial fibrosis through analysis of late gadolinium enhancement (LGE) [8, 9]. This expensive technique may not be available, or may be contraindicated in some patients, especially patients implanted with electronic cardiac devices.

Analysis of the 12-lead electrocardiogram (ECG) can also assess cardiac fibrosis in both ischemic and non-ischemic patients [10, 11].

Finally, biomarkers may be of interest in this population. Galectin-3 (Gal-3) appears to be a promising biomarker of fibrosis [12]. Atrial fibrillation (AF) and HF mechanisms have been associated with high serum Gal-3 levels [13, 14].

We then hypothesized that higher levels of Gal-3, reflecting greater LV fibrotic processes, could predict non-response and outcomes in patients undergoing CRT.

## Methods

### Study protocol

Consecutive patients referred to our department for primary implantation of a CRT defibrillator were prospectively included. Patients had to be over 18 years of age, NYHA class II or III, in sinus rhythm, with a QRS duration ≥ 120 ms, a typical LBBB morphology, and an LVEF still ≤ 35% despite optimal medical therapy for ≥ 6 weeks. Patients with non-LBBB (right bundle branch block or atypical intraventricular conduction delay), atrial fibrillation, contraindications to CMR, and previously implanted pacemaker or cardiac defibrillator were excluded. Patients with non-cardiac conditions usually associated with high Gal-3 levels, such as liver cirrhosis, pancreatitis or chronic inflammatory disease, were also excluded.

The local ethics committee for human research approved the study protocol. All patients signed informed consent before inclusion.

Clinical data including the NYHA functional status, the Minnesota quality of life survey, an ECG and LVEF by trans-thoracic echocardiography (TTE) were collected before device implantation and at 6 months. Late gadolinium enhancement (LGE)-CMR was performed within a month prior to surgery, using a SIEMENS 1.5 T AVENTO machine, after an injection of contrast medium (0.2 mmol/kg of gadotropic acid, Doratem, Guerbet, SA, Villepinte). LGE evaluation was performed blindly by two experienced operators with quantification of fibrosis by two methods: seventeen-segment binary qualitative analysis (0: no LGE; 1: presence of LGE); percentage of LGE in the entire myocardium for quantitative analysis (CVi42 software, Circle Cardiovascular imaging Inc.).

Selvester scoring was also calculated blindly by two trained electrophysiologists, according to the method described by Strauss et al. [11].

TTE was performed within 15 days prior to device implantation and at 6 months. All echographic data were analyzed by a single operator to limit inter-observer variability. The standard parameters were collected: left ventricular end-systolic and end-diastolic volumes (LVESV and LVEDV), LV internal dimension (LVID), Simpson biplane and 3-dimensional LVEF, as recommended [15].

### Galectin-3

The Gal-3 blood test was carried out during the standard preoperative assessment. Determination of Gal-3 level was prospectively completed using the VIDAS Galectin-3 kit (bioMérieux, Marcy-l’Etoile, France). VIDAS galectin-3 is an automated quantitative test. The kit measuring range is 3.3 ng/mL and 100 ng/mL. The assay principle is a one-step immunoassay sandwich method with final fluorescent detection, and has been previously validated in HF patients [16].

### CRT device implantation

The LV pacing lead was implanted transvenously through the coronary sinus in a lateral basal position whenever possible. All patients had biventricular pacing with standardized programming of atrioventricular delay at 120 ms and interventricular delay at 0 ms [17].

### Response to CRT and outcomes

Response to CRT was evaluated by a composite criterion at 6 months: neither death nor hospitalization for major adverse cardiac events (MACE), and the presence of significant LV reverse remodeling (decrease in LVESV ≥ 15%). MACE was defined as hospitalization for HF, cardiogenic shock, and sustained ventricular tachycardia.

Outcomes included death and hospitalizations for MACE.

### Statistical analyses

Analyses were performed using JMP software version 9.0 (SAS Institute Inc., Cary, NC, USA). Numeric data were expressed as mean ± standard derivation (95% confidence interval). Non-parametric tests were performed for comparisons between groups. The predictive parameters of response at 6 months and outcomes were determined by analyzing receiver operating characteristics (ROC) curves to obtain the best cutoff values. The main confounding factors were tested in univariate analysis and parameters significantly associated with response at 6 months (p-value < 0.10), and outcomes were used for analyses in a multivariable Cox model. Survival curves were calculated using the Kaplan–Meier method, and a log-rank test was used to evaluate overall differences between groups. A p-value ≤ 0.05 was considered significant.

## Results

### Characteristics of patients

Sixty-one consecutive patients were prospectively included. Mean age was 61 ± 5 years (28% females), QRS width 163 ± 19 ms and LVEF 27 ± 5%, 41% with CAD (N = 25) (Table 1).

**Table 1:** Baseline characteristics of all patients—responders and non-responders

	All (N = 61)	Responders (N = 49)	Non-responders (N = 12)	p*
Age (years)	61 ± 5	61 ± 5	64 ± 7	0.23
Female sex (%)	17 (28%)	14 (29%)	3 (25%)	0.80
CAD	25 (41%)	18 (37%)	7 (58%)	0.16
Gal-3 (ng/mL)	17 ± 6	16 ± 6	19 ± 8	0.13
CRP (mg/L)	7 ± 11	8 ± 12	6 ± 6	0.53
Baseline Minnesota	30 ± 19	29 ± 18	34 ± 23	0.47
6 months Minnesota	15 ± 14	15 ± 14	16 ± 18	0.90
Baseline NYHA class				
*NYHA II*	28 (46%)	22	6	0.75
*NYHA III*	33 (54%)	27	6	0.75
6 months NYHA class				
*NYHA I*	36 (59%)	36	0	**< ** *0.001*
*NYHA II*	25 (40%)	13	12	**< ** *0.001*
Baseline LVEF (%)	27 ± 5	27 ± 6	27 ± 5	0.91
6 months LVEF (%)	39 ± 8	41 ± 7	32 ± 7	**< ** *0.001*
LVESV (mL/m^2^)	72 ± 26	73 ± 26	69 ± 27	0.65
Presence of LGE	26 (43%)	17 (35%)	9 (75%)	*0.01*
LGE + number of segments	2.2 ± 3.3	1.9 ± 3.4	3.5 ± 2.9	0.15
Percentage of LGE (%)	11 ± 15	8 ± 13	22 ± 16	*0.006*
QRS before CRT (ms)	163 ± 19	163 ± 21	165 ± 13	0.64
QRS after CRT (ms)	135 ± 18	135 ± 20	136 ± 13	0.81
Selvester scoring (%)	17 ± 9	17 ± 9	19 ± 10	0.63
Creatinine clearance (mL/min/1.73 m^2^)	75 ± 23	77 ± 22	71 ± 27	0.40
Diabetes	21 (34%)	14	7	0.06

Significant *p* values are in italics

*A p value ≤ 0.05 was considered significant

Serum Gal-3 level was 16 ± 6 ng/mL with higher rates in CAD patients (19 ± 7 vs 15 ± 5 ng/mL, p = 0.004). Twenty-six (43%) patients presented with LGE (11 ± 15%) (Table 1). Patients with CAD had a higher percentage of LGE as compared with non-ischemic patients (18 ± 17 vs 6 ± 10%, p = 0.002). The lateral basal LV lead position was targeted in 53% of patients (Table 2).

**Table 2:** Position of LV lead

	Basal (N = 32)	Mid (N = 21)	Apical (N = 8)
Antero-lateral (N = 27)	20 (33%)	7 (11%)	0
Lateral (N = 27)	12 (20%)	12 (20%)	3 (5%)
Infero-lateral (N = 7)	0	2 (3%)	5 (8%)

Forty-nine patients (80%) were considered responders to CRT. No significant differences in clinical baseline characteristics were found between responders and non-responders (Table 1). There was no significant difference between CAD and non-ischemic cardiomyopathy (NICM) in response to CRT. Eighteen (72%) patients in CAD group were considered responders at 6 month, versus 31 (86%) patients in NICM group (p = 0.13). At 6 months, LVEF of responders was significantly improved (27 ± 6% up to 41 ± 7% vs 27 ± 5% up to 32 ± 7%, p < 0.001, respectively) and NYHA class was lower in responders group (p < 0.001). Non-responders showed fibrosis on the CMR in 75% of cases versus 35% of responders (p = 0.01). Non-responders showed significantly more LGE on the CMR (8 ± 13% vs 22 ± 16%, p = 0.006). Seven patients presented CAD among non-responders.

All patients have benefited from an optimal medical therapy (Table 3).

**Table 3:** Medications of all patients at baseline—responders and non-responders

	All (N = 61)	Responders (N = 49)	Non-responders (N = 12)	p*
ß-blockers	55 (90%)	45 (92%)	10 (83%)	0.40
ACE or ARB	61 (100%)	49 (100%)	12 (100%)	–
MRA	43 (70%)	36 (73%)	7 (58%)	0.31
Diuretics	46 (75%)	37 (76%)	9 (76%)	0.97
Anticoagulant	6 (10%)	5 (10%)	1 (8%)	0.84
Antiplatelet therapy	38 (62%)	20 (41%)	9 (75%)	0.30

*A p value ≤ 0.05 was considered significant

### Predictive parameters of response to CRT

No clinical or echocardiographic parameter was able to predict response to CRT. The lateral basal LV Lead position did not predict response to CRT (p = 0.83). The presence of fibrosis on the CMR was predictive of response to CRT at 6 months (AUC = 0.74, best cut-off = 14%, sensitivity = 0.73, specificity = 0.75, PPV = 92%, NPV = 41%, p = 0.003) (Table 4). Serum Gal-3 levels were also predictive of response to CRT (AUC = 0.61, best cut-off = 22 ng/mL, sensitivity = 0.84, specificity = 0.42, PPV = 85%, NPV = 38%, p = 0.07). In the multivariable model, a percentage of LGE ≥ 14%, Gal-3 ≥ 22 ng/mL, and diabetes independently predicted non-response (respectively OR = 0.17 [0.03–0.62], p = 0.007; OR = 0.11 [0.02–0.40], p < 0.001; OR = 0.16 [0.04–0.64], p = 0.008).

**Table 4:** Predictive parameters of response at 6 months (Cox model)

	Univariate OR [CI 95%]	p*	Multivariable OR [CI 95%]	p*
Age ≥ 65 year-old	0.42 [0.09–1.99]	0.29		
CAD	0.41 [0.11–1.50]	0.18		
Female sex	1.20 [0.28–5.10]	0.80		
Minnesota ≥ 46	0.42 [0.10–1.82]	0.26		
Gal-3 ≥ 22 (ng/mL)	0.27 [0.07–1.08]	0.07	0.11 [0.02–0.40]	*< * *0.001*
LVEF ≥ 25%	1.03 [0.24–4.43]	0.97		
LVESV (mL/m^2^)	0.59 [0.97–1.02]	0.65		
Presence of LGE	0.18 [0.04–0.74]	*0.01*		
LGE ≥ 14%	0.13 [0.03–0.57]	*0.003*	0.17 [0.03–0.62]	*0.007*
QRS < 150 ms	1.83 [0.20–16.51]	0.57		
Selvester ≥ 24%	0.65 [0.17–2.54]	0.54		
Creatinine clearance (mL/min/1.73 m^2^)	0.99 [0.96–1.02]	0.40		
Diabetes	0.29 [0.08–1.05]	0.06	0.16 [0.04–0.64]	*0.008*

Significant *p* values are in italics

*A p value ≤ 0.05 was considered significant

### Outcomes

The mean follow-up was 37 ± 9 months (range 20–53 months). Only two patients were lost to follow-up. Eight patients were hospitalized for MACE (seven for heart failure, one for ventricular tachycardia); four patients died. Coronary artery disease predicted outcomes (HR = 3.62 [1.07–16.41], p = 0.04) (Table 5). Gal-3 ≥ 22 ng/mL independently predicted long-term outcomes, HR = 3.31 [1.00–11.34], p = 0.05. The percentage of LGE and the Selvester score were not considered significant for outcomes.

**Table 5:** Predictive parameters of long-term outcomes (death and hospitalizations for MACE) (Cox model)

	Univariate HR [CI 95%]	p*	Multivariate HR [CI 95%]	p*
Age ≥ 65 year-old	1.89 [0.29–7.37]	0.45		
CAD	3.62 [1.07–16.41]	*0.04*	2.64 [0.72–12.54]	0.15
Female sex	0.86 [0.19–2.89]	0.82		
Minnesota ≥ 46	0.71 [0.22–2.72]	0.59		
Gal-3 ≥ 22 (ng/mL)	4.33 [1.34–14.01]	*0.02*	3.31 [1.00–11.34]	*0.05*
LVEF ≥ 25%	0.54 [0.17–1.85]	0.31		
LVESV (mL/m^2^)	5.54 [0.53–43.11]	0.14		
Presence of LGE	2.31 [0.73–8.67]	0.16		
LGE ≥ 14%	2.20 [0.70–7.45]	0.17		
QRS < 150 ms	3.20 [0.69–11.31]	0.12		
Selvester ≥ 24%	1.84 [0.49–5.89]	0.34		
Creatinine clearance (mL/min/1.73 m^2^)	0.98 [0.95–1.02]	0.12		
Diabetes	1.24 [0.39–4.68]	0.71		

Significant *p* values are in italics

*A p value ≤ 0.05 was considered significant

**Fig. 1:**
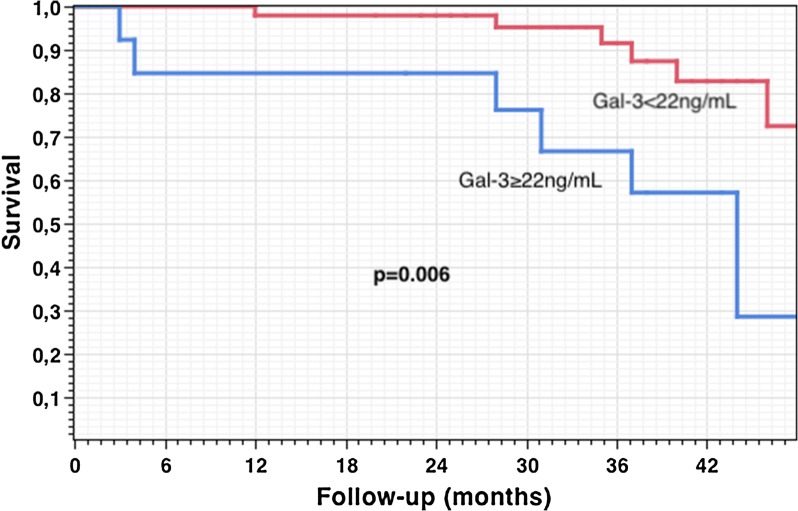
Long-term outcomes after CRT implantation according to serum galectin-3 baseline level

At 48 months, Gal-3 ≥ 22 ng/mL predicted survival after CRT implantation (p = 0.006) (Fig. 1).

## Discussion

### Main results

This is the first study to examine the role of Gal-3, as compared with other parameters of evaluation of fibrosis, in patients undergoing CRT, the cornerstone of electrical treatment in heart failure. Gal-3 appears to be a promising biomarker: it predicts response, i.e. clinical status and hemodynamic improvement, after CRT, as well as long-term outcomes.

### Galectin-3

Gal-3 is the only chimera-type member of family of ß-galactoside-binding animal lectins.

Some clinical studies have confirmed the predictive value of Gal-3 for all-cause mortality in HF patients [18, 19]. Patients with Gal-3 level > 17.8 ng/mL have been shown to have a higher risk of mortality and HF hospitalization [20].

In an animal model, rats treated with intra-pericardial administration of low levels of Gal-3 developed HF, characterized by a significant decrease in LVEF. In fact, the density of LV collagen type I was threefold in Gal-3 treated rats, whereas no variation for type III deposits was observed [21]. This increased collagen type I/III ratio increased myocardial rigidity; whereas collagen III forms an elastic network storing kinetic energy as elastic recoils, collagen I represents a stiff fibrillary protein providing tensile strength. On the other hand, constitutive inactivation of the Gal-3 gene (mouse gal−/−) protected against the development of such abnormalities, highlighting the involvement of this lectin in this progressive myocardial fibrotic invasion. Gal-3 then appears as a major mediator of fibrogenesis, contributing to HF development and progression.

### Patient selection

The current literature shows between 40 and 60% of responders to CRT depending on the criteria used [1]. The high rate of an 80% response to CRT in our study may be explained by patient screening. Typical LBBB morphology and large QRS width are known to be associated with an improved response to CRT [22, 23]. Only patients with typical LBBB morphology were included (mean QRS duration was 163 ms).

In MADIT CRT study, typical LBBB morphology and QRS duration ≥ 150 ms were also identified as predictive factors [3, 24]. Patient selection is the key to improving response to CRT [25].

### Fibrosis and CRT

This study confirms the need for fibrosis assessment before CRT [9]. Extensive myocardial fibrosis is indeed associated with lower response and subsequent higher morbidity and mortality in this population [22, 23]. Chalil et al. described that transmurality ≥ 51%, posterolateral location, and complete myocardial fibrosis ≥ 33% were predictive of non-response [8]. CMR with LGE analysis should be performed before any CRT implantation. An extent of fibrosis ≥ 14% was indeed associated with non-response in our study. This lower cut-off may be explained by a better selection of patients who are more likely to respond. Gal-3, as a simple biomarker, may help to select patients requiring CMR with more precise analysis through LGE measurement, as it correlated with the presence of significant LGE.

Patients with higher levels of Gal-3 are also at higher risk of non-response and worse outcome. These patients require close monitoring and accurate CRT optimization. CMR evaluation in these patients appears mandatory in order to position the LV pacing lead in a region free from scarring [24–26]. However, patients with a low serum Gal-3 level theoretically have sparse fibrosis or none at all and are likely to respond to CRT. CMR appears optional in these patients.

Gal-3 may be used as a prognostic marker in HF patients, especially in patients undergoing CRT implantation. In the CARE-HF trial, a Gal-3 > 30 ng/mL was associated with increased HF hospitalization and mortality rates [27].

## Limitations

The Gal-3 level often increases in renal failure and chronic inflammatory diseases. These patients were excluded so that no significant difference in renal clearance and CRP between groups was present in our study. Patients without typical LBBB morphology were also excluded. Even if these patients tend to respond less to CRT, it could also be useful to evaluate Gal-3 in this population.

Response to CRT depends on many different parameters. A role of LV lead position, percentage of biventricular pacing, loss of LV capture, compliance to medical therapy or atrial fibrillation cannot be ruled out.

## Conclusions

Serum galectin-3 levels predict response and long-term outcomes after cardiac resynchronization therapy in chronic heart failure patients and may help select patients requiring closer monitoring.

## Abbreviations

HF: heart failure
CRT: cardiac resynchronization therapy
ESC: European Society of Cardiology
LV: left ventricular
CMR: cardiac magnetic resonance
LGE: late gadolinium enhancement
ECG: electrocardiogram
Gal-3: galectin-3
AF: atrial fibrillation
NYHA: New-York Heart Association functional status
LBBB: left bundle branch block
LVEF: left ventricular ejection fraction
TTE: trans thoracic echography
LGE: late gadolinium enhancement
LVESV: left ventricular end-systolic volume
LVEDV: left ventricular end-diastolic volume
LVID: left ventricular internal dimension
MACE: major adverse cardiac events
CAD: coronary artery disease
ROC: receiver operating characteristics
NICM: non-ischemic cardiomyopathy
CRP: C reactive protein
ACE: angiotensin-converting enzyme
ARB: angiotensin receptor blocker
MRA: mineralocorticoid receptor antagonist
AUC: area under curve
PPV: positive predictive value
NPV: negative predictive value
OR: odds ratio
CI: confidence interval
HR: hazard ratio
